# Label-free Electrochemiluminescent Immunosensor for Detection of Prostate Specific Antigen based on Aminated Graphene Quantum Dots and Carboxyl Graphene Quantum Dots

**DOI:** 10.1038/srep20511

**Published:** 2016-02-04

**Authors:** Dan Wu, Yixin Liu, Yaoguang Wang, Lihua Hu, Hongmin Ma, Guoqin Wang, Qin Wei

**Affiliations:** 1 Key Laboratory of Chemical Sensing & Analysis in Universities of Shandong, School of Chemistry and Chemical Engineering, University of Jinan, Jinan 250022, China

## Abstract

Prostate-specific antigen (PSA) was used as the model, an ultrasensitive label-free electrochemiluminescent immunosensor was developed based on graphene quantum dots. Au/Ag-rGO was sythsized and used as electrode material to load a great deal of graphene quantum dots due to the large surface area and excellent electron conductivity. After aminated graphene quantum dots and acarboxyl graphene quantum dots were modified onto the electrode, the ECL intensity was much high using K_2_S_2_O_8_ as coreactant. Then, antibody of PSA was immobilized on the surface of modified electrode surface through the adsorption of Au/Ag toward proteins, leading to the decrease of the ECL intensity. As proven by ECL spectra test and electrochemical impedance spectroscopy (EIS) analysis, the fabrication process of the immunosensor is successful. Under the optimal conditions, the ECL intensity decreased linearly with the logarithm of PSA concentration in the range of 1 pg/mL ~ 10 ng/mL. The detection limit achieved is 0.29 pg/mL. The immunosensor results were validated through the detection of PSA in serum samples with satisfactory results. Due to excellent stability, high sensitivity, acceptable repeatability and selectivity, the immunosensor has promising applications in disease and drug analysis.

Immunosensors based on antibody-antigen binding are one of the most widely used to detect disease related substances, which are known as biomarkers, in clinical diagnostics^1^. Prostate cancer is the most common malignancy diagnosed in men and the second leading cause of cancer death for men in the United States^2^. Prostate-specific antigen (PSA), an extracellular serine protease which belongs to the kallikrein family, remains most commonly used biomarker for prostate cancer. Measurement of PSA level is not only used for the early detection of the cancer but also to monitor the response of the patients to the therapy^3^. For this reason, the quantitative analysis of serum PSA level with ultra-sensitivity facilitates an early detection of prostate cancer and also recurrence of the disease.

There are two types of immunosensors. One is sandwich-type immunosensors and the other is label-free immunosensors. Compared with sandwich-type immunosensor, label–free immunosensors have some other advantages, for instance, they could be directly used to monitor the binding process of antibody–antigen reaction and overcome the condition limitation of enzymes. Therefore, the label–free immunosensors has become a remarkable analytical tool for detection of biomolecules. Many immunosensors for the determination of PSA are continuously being put forward based on electrochemistry, fluorescence, electrochemiluminescence (ECL) and so on^4^,^5^,^6^,^7^,^8^,^9^. Among them, ECL has been proven to be a useful detection method with high sensitivity and selectivity owing to the combination advantages of chemiluminescence and electrochemistry. Thus, ECL-based label-free immunosensors was developed owing to the integrated advantages of ECL and immunosensors with high specificity and affinity in this work.

Graphene quantum dot (GQD), a class of carbon nanomaterials containing one or few layered sheets with lateral dimension less than 100 nm, have several unique properties^10^. Due to the low toxicity, biocompatibility, and photostability, GQD has many applications in those fields such as cell imaging, drug carrier, biosensors and so on^11^,^12^,^13^,^14^,^15^,^16^,^17^. In this report, GQD was used as the ECL reagent to construct a label-free ECL sensors for PSA. Au/Ag-rGO was synthesized and used as electrode material to load a great deal of aminated graphene quantum dots and carboxyl graphene quantum dots. Then, antibody of PSA was immobilized on the surface of modified electrode surface through the adsorption of Au/Ag toward proteins, leading to the decrease of the ECL intensity. The decreased intensity is proportional to the logarithm of PSA concentration. The ECL immunosensor features high sensitivity, good selectivity and wide linear range.

## Experimental

### Materials and reagents

Aminated graphene, aminated graphene quantum dots and carboxyl graphene quantum dots were purchased from Nanjing XFNANO Materials Tech Co., Ltd. (China). The PSA and corresponding antibody were purchased from Beijing Dingguo Changsheng Biotechnology Co. Ltd. (China). AgNO_3_, HAuCl_4_ and bovine serum albumin (BSA, 96–99%) were purchased from Sigma-Aldrich (Beijing, China). 1-ethyl-3-(3dimethylamino-propyl) carbodiimide (EDC), N-hydroxysuccinimide (NHS), sodium citrate and K_2_S_2_O_8_ were obtained from Shanghai Aladdin Chemistry Co., Ltd, China. All other chemicals were of analytical grade and used without further purification.

### Apparatus

ECL measurements were carried out by using the MPI-F flow-injection chemiluminescence detector (Xi′an Remax Electronic Science Tech. Co. Ltd., China). Electrochemical measurements were operated by employing a CHI760D electrochemical workstation (Chenhua Instrument Shanghai Co., Ltd., China). A conventional three-electrode system was used including a platinum wire electrode as the auxiliary electrode, a saturated calomel electrode (SCE) as the reference electrode and a glassy carbon electrode (GCE, 4 mm in diameter) as the working electrode. The scanning electron microscope (SEM) images were obtained by the field emission SEM (ZEISS, Germany).

### Synthesis of Au/Ag-rGO

Au/Ag-rGO was prepared using the following procedure. 80 mg aminated graphene powder was dispersed in 50 mL ultrapure water and under continuous ultrasound for 10 h. Then, 40 mg AgNO_3_, 5 mL 1% HAuCl_4_ and 5 mg PVP were added into the above solution. 4 mL 50 mM sodium citrate was added dropwise to reduce AgNO_3_ and HAuCl_4_ after 6 h of stirring. After 24 h of stirring, the Au/Ag-rGO suspension was filtered and then dried at 35 °C in vacuum.

### Preparation of Au/Ag-rGO/Aminated-GQDs/Carboxyl-GQDs

Aminated graphene quantum dots (5 mg/mL, 1 mL), carboxyl graphene quantum dots (5 mg/mL, 1 mL), 40 mg EDC and 20 mg NHS were mixed into a centrifuge tube and stirred for 5 h at 4 °C. Au/Ag-rGO/Aminated-GQDs/Carboxyl-GQDs were prepared by adding 5 mg of Au/Ag-rGO into the above Aminated-GQDs/Carboxyl-GQDs solution and incubating for 24 h at 4 °C. After that, the solution was centrifugated and the products were redispersed in 1 mL of PBS (pH 7.4) and stored at 4 °C for use. The final concentration of Au/Ag-rGO is 5 mg/mL.

### Modification of electrodes

**Figure 1:**
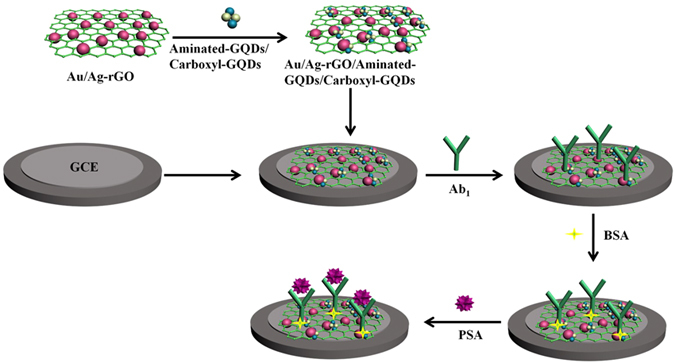
The illustration of ECL immunosensor fabrication process.

The illustration of ECL immunosensor fabrication process is depicted in Fig. 1. A GCE was polished until a mirrorlike surface appeared and cleaned thoroughly before use with the sequential use of 1.0, 0.3, and 0.05 μm alumina powder. After rinsing with ultrapure water, 6 μL of Au/Ag-rGO/Aminated-GQDs/Carboxyl-GQDs was dropped on the surface of the GCE and dried, followed by modification 6 μL of 10 μg/mL PSA antibody. After rinsing, the unreacted active sites on the electrode surface were deactivated by 3 μL of 1% bovine serum albumin (BSA) solution for 1 h. Finally, the electrode was incubated with different concentration of PSA for 30 min at 4 °C and then washed with buffer solution to remove the excess CEA. Thus, the ECL immunosensor was fabricated completely and was ready to be used.

## Results and Discussion

### Characterization of Au/Ag-rGO

**Figure 2:**
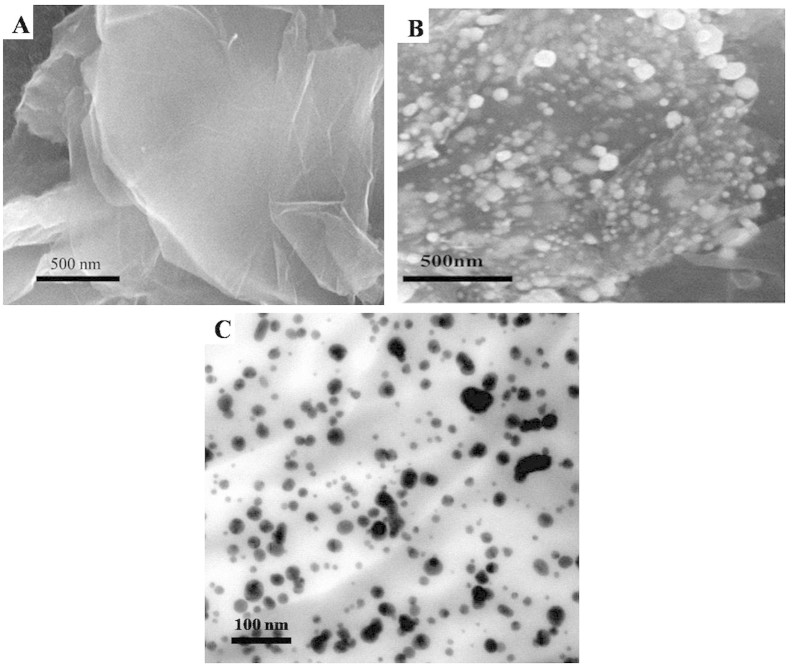
SEM of rGO (**A**) and Au/Ag-rGO (**B**), TEM of Au/Ag-rGO/Aminated-GQDs/Carboxyl-GQDs (**C**).

Figure 2A,B show the SEM images of rGO and Au/Ag-rGO. Obviously, large amounts of Au/Ag NPs were distributed on the surface of Au/Ag-rGO, which indicated that the Au/Ag-rGO were obtained as expected. Au/Ag-rGO was used as electrode material to load a great deal of graphene quantum dots due to the large surface area and excellent electron conductivity. Figure 2C shows the TEM image of Au/Ag-rGO/Aminated-GQDs /Carboxyl-GQDs and Au/Ag NPs and GQDs were uniformly dispersed on the surface of rGO.

### Characterization of the immunosensor

**Figure 3:**
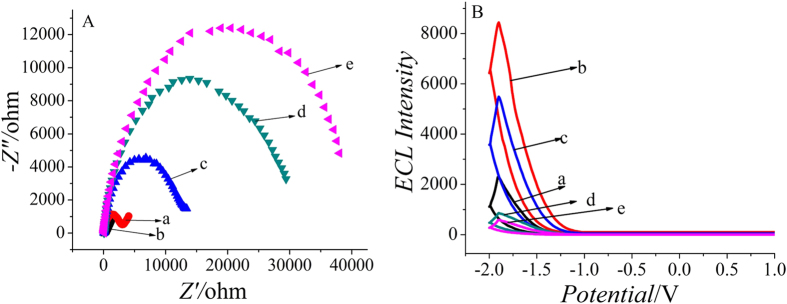
EIS in the presence of 5.0 mmol/L [Fe(CN)_6_]^3−/4−^ solution containing 0.1 mol/L KCl (**A**) and ECL intensity–potential curves in PBS containing 100 mmol/L K_2_S_2_O_8_ with the potential range of −2.0 to 0 V (**B**). (a) bare GCE (b) Au/Ag-rGO/Aminated-GQDs/Carboxyl-GQDs/GCE (**c**) PSA antibody/Au/Ag-rGO/Aminated-GQDs/Carboxyl-GQDs/GCE (d) BSA/PSA/Au/Ag-rGO/Aminated-GQDs/Carboxyl-GQDs/GCE (e) PSA/BSA/PSA antibody /Au/Ag-rGO/Aminated-GQDs/Carboxyl-GQDs/GCE.

Electrochemical impedance spectroscopy (EIS), a tool for evaluating electron transfer resistance, was used to characterize the stepwise modification of the electrodes^18^,^19^. The EIS curves of different fabrication steps in 5.0 mmol/L [Fe(CN)_6_]^3−/4−^ solution containing 0.1 mol/L KCl are displayed in Fig. 3A. Non-modified GCE showed a small semicircle diameter (curve a), implying a low electron transfer resistance. After Au/Ag-rGO/Aminated-GQDs/Carboxyl-GQDs were modified on the electrode, there was a decrease in semicircle diameter (curve b) which might be attribute to the excellent conductivity of Au/Ag-rGO. The sequential immobilization of PSA antibody (curve c), BSA (curve d) and PSA (curve e) led to gradual increase of the electron transfer resistance due to the insulation properties of protein.

In order to further prove that the electrode was modified successfully, the stepwise fabrication process of the immunosensor was also characterized by ECL, as shown in Fig. 3B. Compared with the bare electrode (curve a), the ECL response was enhanced greatly after Au/Ag-rGO/Aminated-GQDs/Carboxyl-GQDs was immobilized on it (curve b), suggesting that Aminated-GQDs and Carboxyl-GQDs have good ECL properties. Subsequently, a gradual decrease in the ECL signal was achieved when PSA antibody (curve c), BSA (curve d) and PSA (curve e) was modified onto the electrode surface successively, which could be attributed to the block of biomacromolecules hindering the electron transfer. Both the above results were consistent with the fact that the electrode was modified as expected.

### Optimization of experimental conditions

**Figure 4:**
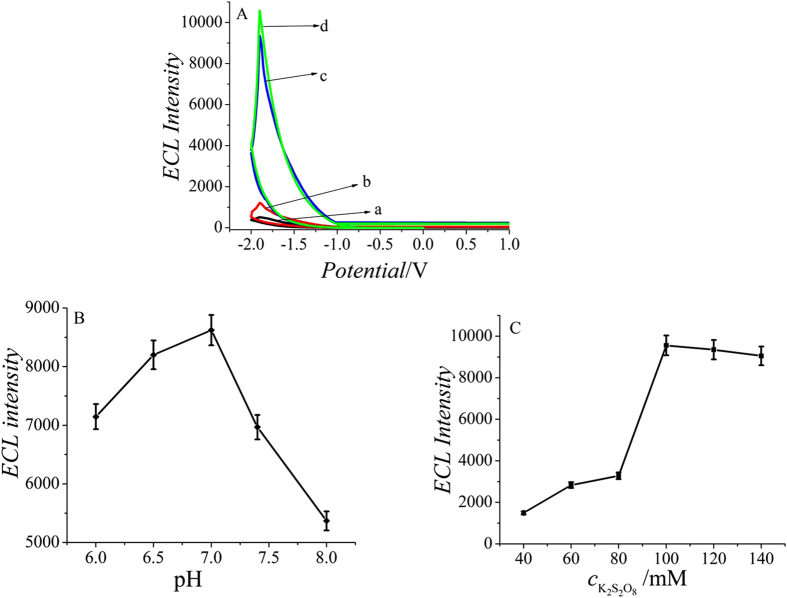
ECL intensity–potential curves (**A**), effect of pH (**B**) and the concentration of K_2_S_2_O_8_ (**C**) on the ECL intensity. (a) bare GCE (b) Aminated-GQDs (2.5 mg/mL)/GCE (c) Carboxyl-GQDs (2.5 mg/mL)/GCE (d) Aminated-GQDs (2.5 mg/mL)/Carboxyl-GQDs (2.5 mg/mL)/GCE.

As shown in Fig. 4A, compared with the bare electrode (curve a), the ECL response was enhanced after GCE was modified. When both aminated-GQDs and carboxyl-GQDs were modified onto the electrode, the ECL intensity was higher (curve d) than that of aminated-GQDs (curve b) and carboxyl-GQDs (curve c), respectively. This is the reason why both aminated-GQDs and carboxyl-GQDs were chosen.

To obtain an optimal ECL signal, pH value of substrate solution was investigated. Keeping the concentrations of TB constant, the effect of pH on the ECL intensity was studied over a pH range from 6.0 to 8.0, as shown in Fig. 4B. The ECL intensity increased with the increase of pH from 6.0 to 7.0 and reached the maximum. After that, the ECL intensity decreased accordingly with pH increasing from 7.0 to 8.0. Therefore, pH 7.0 was chosen as the optimal value for ECL response.

In acidic solution, the reduction of proton to hydrogen would take place at the applied negative potential, which might inhibit the reduction of S_2_O_8_^2−^. However, the intermediate SO_4_^•−^ from S_2_O_8_^2−^ reduction would be scavenged by OH^•−^ leading to a decrease in the ECL intensity in basic solution^20^. The effect of coreactant K_2_S_2_O_8_ concentration in the substrate solution on ECL intensity was also tested. As shown in Fig. 4C, the ECL intensity increased when K_2_S_2_O_8_ concentration increased from 40 to 100 mmol/L because more GQDs^*^ is produced from oxidation of GQDs^•−^ by the electrogenerated SO_4_^•−^ and remained constant in the range 100 ~ 140 mmol/L. Thus, 100 mmol/L K_2_S_2_O_8_ was chosen for further study in order to ensure an adequate sensitivity and save the dosage.

**Figure 5:**
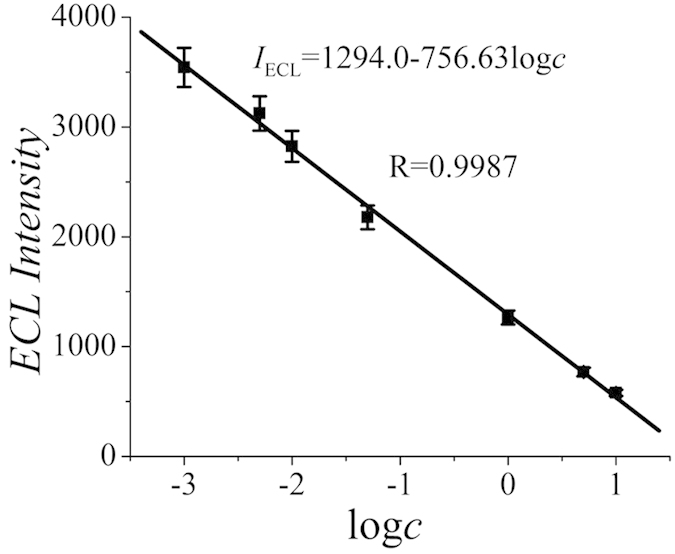
Calibration curve of the immunosensor for different concentrations of PSA.

The quantitative determination under the optimal conditions was carried out on the modified GCE incubated with different concentrations of PSA. As can be display in Fig. 5, a linear dependence between the ECL signals and the logarithm of PSA concentration was obtained in the range from 1 pg/mL to 10 ng/mL with a detection limit of 0.29 pg/mL (S/N = 3). The linear regression equation was *I*_ECL_ = 1294.0 − 756.63 lg*c*. Compared with the other immunosensors previously reported for the detection of PSA (as shown in Table 1), the proposed ECL immunosensor has a lower or comparable detection limit.

The ECL process could be expressed as following:


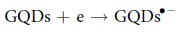



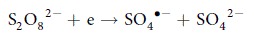



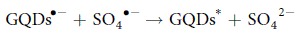



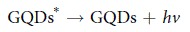


Upon potential scanning with an initial negative direction, the GQDs were reduced to GQDs^•−^, while the coreactant S_2_O_8_^2−^ was reduced to the strong oxidant SO_4_^•−^, and then GQDs^•−^ could be oxidized by SO_4_^•−^ to generate the excited state GQDs^*^, leading to emit light.

### Stability and Selectivity

**Figure 6:**
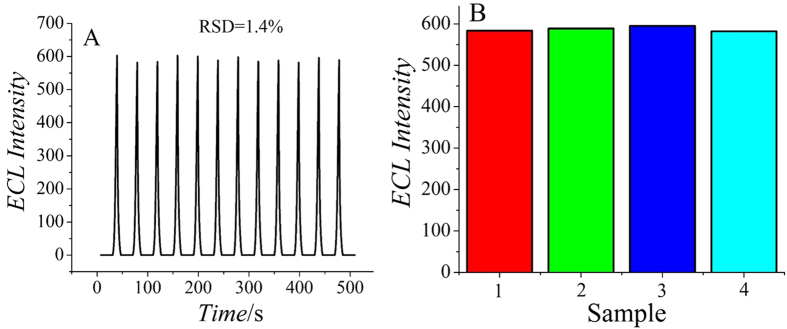
The stability of the immunosensor incubated with 5 ng/mL PSA under continuous potential scanning for 12 cycles (**A**) and the selectivity of the immunosensor (**B**). 5 ng/mL PSA (1), 100 ng/mL CEA + 5 ng/mL PSA (2), 100 ng/mL BSA + 5 ng/mL PSA (3), 100 ng/mL glucose + 5 ng/mL PSA (4).

The stability of the ECL immunosensor was estimated under continuous potential scans for 12 cycles for the detection of PSA (5 ng/mL). As shown in Fig. 6A, there was little change of the ECL intensity and the relative standard deviation (RSD) was 1.4%, supporting the good stability of the proposed immunosensor. The storage stability of the proposed immunosensor was also studied by storing it at 4 °C when not in use, and the results were satisfactory because after 3 days of storage the immunosensor retained 96.9% of its initial response and its response decreased to 90.7% after 5 days.

Figure 6B shows the selectivity of the ECL immunosensor for PSA. The ECL responses were measured by mixing 5 ng/mL of PSA with 100 ng/mL carcinoembryonic antigen (CEA), 100 ng/mL BSA and 100 ng/mL glucose, respectively. The ECL intensity exhibited no obvious change, which illustrated excellent selectivity and specificity of the ECL immunosensor for PSA.

### Serum sample analysis

The amount of PSA was measured 5 times in human serum sample and the relative standard deviation (RSD) was calculated to obtain the precision. The accuracy was also studied through a recovery experiment using standard addition method. An appropriate amount of PSA standard solution was added to corresponding samples. With the same experiments measured for five times, the average recovery was calculated. It can be found from Table 2 that the relative standard deviation is 4.6% and the recovery is 100.1%. The results indicate that the proposed immunosensor has important application value in clinical research and diagnosis.

## Conclusions

This work demonstrated an ultrasensitive electrochemiluminescent immunoassay based on graphene quantum dots. The success of this work relied on the use of Au/Ag-rGO to assemble quantities of aminated graphene quantum dots and carboxyl graphene quantum dots, thus making ECL signal amplification. Great amplification together with the specificity of immunoreaction made sensitive detection of PSA possible. The proposed immunosensor achieved excellent performance with high sensitivity, good selectivity and stability. On one hand, this type of electrochemiluminescent immunosensor should be feasible for the field of cancer biomarker diagnosis and other life science fields. And on the other hand, this principle could be also applied to many other bioassays.

## Additional Information

**How to cite this article**: Wu, D. *et al*. Label-free Electrochemiluminescent Immunosensor for Detection of Prostate Specific Antigen based on Aminated Graphene Quantum Dots and Carboxyl Graphene Quantum Dots. *Sci. Rep.* **6**, 20511; doi: 10.1038/srep20511 (2016).

## Figures and Tables

**Table 1:** Comparisons of proposed method with other reported immunosensors for PSA.

Electrode materials	Linear range	Detection limit	Reference
Au-NS/ POEGMA-co-GMA brush	5 pg/mL ~ 1000 ng/mL	2.3 pg/mL	21
MWCNTs/IL/TH	0.2 ~ 1.0 ng/mL 1.0 ~ 40 ng/mL	20 pg/mL	22
SWNTs	—	0.25 ng/mL	23
AuNP-PANI/Au	1.0 pg/mL ~ 100 ng/mL	0.6 pg/mL	24
Au/Ag-rGO/Aminated-GQDs/Carboxyl-GQDs	1.0 pg/mL ~ 10 ng/mL	0.29 pg/mL	This work

**Table 2:** Results for the determination of PSA in human serum sample.

Sample	Content of PSA (ng/mL)	Average (*n* = 5, ng/mL)	RSD (%)	Added (ng/mL)	Recovery value (ng/mL)	Recovery (*n* = 5, ng/mL)
Human serum	5.30	5.61	4.6	2.50	2.52	100.1
	5.76			2.50	2.57	
	5.49			2.50	2.39	
	5.97			2.50	2.61	
	5.52			2.50	2.42	
